# Determinants of sleep quality and their impact on health outcomes: a cross-sectional study on night-shift nurses

**DOI:** 10.3389/fpsyt.2024.1506061

**Published:** 2024-12-24

**Authors:** Qingqing Xiao, Xia Huang, Tao Yang, Lei Huang, Nan Li, Jingjun Wang, Junqiang Huang, Yalin Huang, Hao Huang, Ya Wang

**Affiliations:** ^1^ Mental Health Center, West China Hospital, Sichuan University, Chengdu, Sichuan, China; ^2^ West China School of Nursing, Sichuan University, Chengdu, Sichuan, China; ^3^ School of Nursing, Xinxiang Medical University, Xinxiang, China; ^4^ West China Tianfu Hospital, Sichuan University, Chengdu, Sichuan, China; ^5^ West China Hospital, Sichuan University, Chengdu, Sichuan, China; ^6^ Department of Psychiatry, Dekang Hospital, Chengdu, Sichuan, China; ^7^ Department of Nursing, West China Hospital, Sichuan University, Chengdu, Sichuan, China

**Keywords:** night-shift nurses, sleep quality, influencing factors, chronic fatigue, cross-sectional study

## Abstract

**Purpose:**

This study aimed to identify determinants of sleep quality and explore their adverse health outcomes among night-shift nurses in China.

**Method:**

Through convenience sampling, this cross-sectional study enrolled a total of 711 night-shift nurses aged 20-55 years who completed questionnaires from which details regarding their sociodemographic characteristics, health-related indicators and sleep quality based on the Pittsburgh Sleep Quality Index (PSQI) were extracted. A generalized linear regression analysis was then created to identify factors influencing sleep quality. Pearson correlation analysis was used to analyze the relationship between sleep quality and chronic fatigue.

**Results:**

The prevalence rate of poor sleep quality among night-shift nurses reached as high as 90.1%. Our results showed that education level, years of working experience, quality of make-up sleep before and after night shifts, daily routine and diet were the primary factors affecting sleep quality (*p* < 0.01). Nurse fatigue had a significant positive correlation with subjective sleep quality, sleep latency, sleep duration, sleep disturbance, medications to sleep, daytime dysfunctions and global sleep quality (*p* < 0.01). However, nurse sleep efficiency had a significant negative correlation with fatigue (*p* < 0.01).

**Conclusions:**

Higher education level, longer working years, worse quality of make-up sleep before and after night shifts, daily routine and daily diet were risk factors for poor sleep quality among nurses. Poor sleep among night-shift nurses is strongly correlated with chronic fatigue.

## Introduction

Hospitals are institutions that provide healthcare services 24 h a day, 7 days a week, with nurses largely being locked into schedules including a night shift (1). Based on the classification of Chinese hospitals, tertiary and grade A hospitals are tasked to service a large number of patients; unfortunately, bed-to-nurse ratios have remained unsatisfactory (2). Nursing shortages and heavy clinical nursing workloads force almost all nurses to undertake night shift work from the beginning of their nursing careers (3). Frequent night shifts among clinical nurses cause disturbances in sleep mechanisms to which adaptation is difficult (3). Sleep experts identify two major stages of wakefulness and sleep, with sleep further subdivided into light sleep, deep sleep, and rapid eye movement (REM) behavior, and good sleep quality is characterized by the deep sleep stage occupying a relatively high proportion of the sleep duration (4). Night shift work can disrupt the cycles of cortisol and melatonin, shorten deep sleep time, lead to sleep deprivation, and directly affect sleep quality (5, 6).

A study from China that surveyed 3,206 nurses found that night-shift nurses demonstrated relatively worse sleep quality (55.1%) and more health problems (20.7%) than did those in other shifts (7). A 2-year follow-up retrospective analysis in the National Nurse Health Study showed that higher night shift load was associated with more sleep problems, such as shortened sleep duration, poor sleep quality and sleep deprivation (6). According to a study from Saudi Arabia, nurses who worked night shifts had poorer quality sleep than their counterparts who worked day shifts (8). Another study from South Korea showed a high prevalence (79.8%) of poor sleep quality among nurses, which may lower their productivity (9). A systematic review and meta-analysis on 29 studies, which included 3,935 fixed day-shift nurses, 3,777 shift nurses and 1,559 fixed night-shift nurses (10) revealed that nurses working rotating shifts and fixed night shifts had poorer sleep quality than did those working fixed day shifts (10). However, a study in the United States found no significant differences in sleep quality between nurses working day and night shifts (11).

Unlike day shifts, night shifts have been associated with poor physical and mental health through its adverse effects on sleep, circadian rhythms and dietary and beverage consumption, along with impaired cognitive function that increases nurse errors (12). A previous study showed that nurses working night shifts showed poorer sleep quality and nearly twofold increased risk of depression than did those working day shifts (1). Decreased sleep quality can promote a range of physical health issues, such as headaches, gastrointestinal discomfort, obesity, diabetes, hypertension, coronary heart disease and even breast cancer (13). Additionally, research has shown that compared to day-shift nurses, night-shift nurses are more likely to experience psychological and emotional problems, such as occupational stress and anxiety (14, 15). Indeed, a study from Chongqing, which surveyed nurses from 18 tertiary hospitals and 16 secondary hospitals, found that a night-shift frequency of 3 to 4 times per month, night-shift durations of 9-12 h, sleep time delay of ≥2 h after night shift and total sleep time of <4 h after night shift were shift-related factors that increased the levels of occupational stress and anxiety (16).

A systematic review and meta-analysis study showed that adult chronic fatigue syndrome patients spend longer time in bed, longer sleep onset latency, longer awake time after sleep onset, decreased sleep efficiency (17). However, there are few studies exploring the mutual influence between sleep and fatigue among night shift nurses. In the southwestern region of China, mainly in Sichuan Province, Yunnan Province, and Guizhou Province, the economic development lags behind that of coastal cities, the distribution of medical resources is uneven (18), and nurses generally face high work pressure. Limited epidemiological studies have evaluated sleep problems among clinical nurses in southwest China, but the research on influencing factors is not comprehensive. This study therefore aimed to identify factors determining sleep quality among night-shift nurses in southwest China and explore the relationship between sleep quality and chronic fatigue among night-shift nurses.

## Methods

### Participants

This cross-sectional study recruited 881 nurses via convenience sampling from 18th March, 2023 to April 13th, 2024 in the Sichuan in China. Participants satisfied the following criteria: licensed registered nurse; had clinical night-shift experience; age above 18 years old; and participated voluntarily. The exclusion criteria were as follows: nursing interns; and nurses who had been on sick leave for more than three months. This study was performed in accordance with the ethical principles stated in the Declaration of Helsinki and was approved by the Biomedical Research Ethics Committee, West China Hospital of Sichuan University (No.1581).

### Measures

#### General information

A self-administered questionnaire was established to collect sociodemographic data (e.g. gender, age, marriage, educational level, working life, professional qualifications and average monthly income), night shift data (e.g. average duration of each night shift, number of consecutive days per night shift, average weekly night shift frequency, average night shift interval days, quality of make-up sleep the day before night shift, quality of make-up sleep the day after night shift and night shift in life) and lifestyle data (e.g. daily routine, daily exercise and daily diet).

### Scale evaluation

#### Pittsburgh Sleep Quality Index—Chinese Version (PSQI-C)

The PSQI-C scale, which was translated into Chinese and revised by Liu et al. (19) in 1996, is a valid instrument used to measure sleep quality and patterns. The PSQI is a 19-item self-reported questionnaire used for evaluating sleep quality over the previous month. The 19 questions are combined into 7 clinically-derived component scores comprising sleep quality, sleep latency, sleep duration, habitual sleep efficiency, sleep disturbance, use of sleep medication and daytime dysfunction. Each component is scored on a 4-point Likert-type scale (0, 1, 2 and 3) and weighted equally from 0 to 3. The 7 component scores are then summed to obtain a global score ranging from 0 to 21, with higher scores indicating worse sleep quality (20). A total PSQI score of 7 or more suggests the presence of sleep disorder (21). In most studies, the Cronbach’s alpha coefficient of the PSQI score falls between 0.70 and 0.83, showing good internal consistency (22).

#### Brief Fatigue Inventory-Chinese version (BFI-C)

This scale is divided into two parts (23). The first part measures the degree of comprehensive fatigue, adopting a 0-10 grading method according to the degree of present fatigue, the most fatigue experienced in the past 24 h and the usual fatigue experienced. The average value of the total score is taken as the comprehensive fatigue degree score. The second part measures the influence of fatigue on general activity, mood, walking ability, daily work (including work outside and housework), relationships with others and enjoyment of life. Higher scores on this scale indicate higher fatigue levels, with 7 points and above indicating severe fatigue.

### Data collection

The questionnaire survey was conducted by Wen Juan Xing (www.wjx.cn), an online data collection platform. To ensure data accuracy, all items were set as required questions to ensure the completeness of the questionnaires and each IP address or device was limited to submit the questionnaires only once to avoid repetition. Researchers initially contacted the nursing department directors of the participating hospitals before data collection, explicated the objectives and some details regarding the study and invited them to advertise the study among nurses employed in their hospitals. Afterwards, we sent the link to the online anonymous questionnaires to the nursing department directors of each hospital through WeChat (a popular social media platform with over 1 billion active users in China). The nursing department directors then forwarded the link to their respective nurses via private WeChat groups. An explanatory statement, outlined at the head of the questionnaires, was provided for the purpose of informed consent. The questionnaires can be completed in approximately 10-15 min. Data from Wen Juan Xing were imported into Excel for screening. A total of 881 nurses clicked on the link to fill out the questionnaire. After excluding questionnaires with some missing items (143 nurses in the questionnaire stated that they did not work night shifts) and a high proportion of consistent responses (27 questionnaires have completely consistent options in the testing section of the scale), 711 valid questionnaires were ultimately obtained.

### Statistical analysis

General information was expressed as frequencies and percentages (%) and PSQI-C and BFI-C scores were described as means ± standard deviations (M ± SD). The independent sample t-test and one-way analysis of variance (ANOVA) were used to compare differences in sleep quality among nurses according to their general characteristics. The Pearson’ s correlation coefficient was computed to examine the association between sleep quality and fatigue. Moreover, generalized linear regression analysis was applied to identify factors influencing sleep quality. All statistical tests were performed using SPSS version 21.0 (IBM Corp., Armonk, NY, USA) and were two-sided, with a *p* value of <0.05 indicating statistical significance.

## Results

### Participant characteristics

The results of this article show that 642 out of 711 night shift nurses have poor sleep quality, with a prevalence rate of 90.1%. **Table 1** displays the sociodemographic characteristics of night-shift nurses. The mean age of the participants was 29.52 years (SD = 5.29), ranging from 20 to 55 years. The majority of the 711 nurses were female (n = 646, 90.9%), under 40 (n = 677, 95.2%), unmarried (n = 315, 44.3%), had graduated from college (n = 501, 70.5%) and had primary professional qualifications (n = 501, 80.3%). Moreover, 340, 132, 215 and 24 nurses had <5 years, 6-9 years, 10-19 years and over 20 years of work experience, respectively. The average monthly income of most nurses (n = 427, 60.1%) was between 5,000 and 10,000 yuan. The average night shift duration was <8, 9-16 and >17 h for 480, 260 and 25 nurses, respectively. Most nurses (n = 407, 57.2%) worked two night shifts in a row, with 346 nurses (48.7%) having an average night-shifts frequency of two per week. A total of 413 nurses (58.1%) had an average night shift interval of <5 days. Around one sixth of the nurses believed that they had poor quality of make-up sleep during night shifts. More than half of the nurses (n = 367, 51.6%) had worked night shifts for over 5 years. Among the included nurses, 122 (17.2%) believed that their daily routines were extremely irregular, 393 (55.3%) had a habit of exercising every day and 57 (8.0%) believed that their daily diet was extremely irregular.

**Table 1:** Characteristics of night-shift nurses and univariate analysis of poor sleep quality with different demographic characteristics (n=711).

Variables	Group	n (%)	PSQI score (Mean MeSD)	t/F	P value
General demographic factors					
Gender	Male	65 (9.14)	9.62 ± 3.035	-2.234	0.026
	Female	646 (90.86)	10.52 ± 3.132		
Age	18~29	421 (59.21)	10.30 ± 3.026	6.682	0.001
	30~39	256 (36.01)	10.42 ± 3.180		
	≥40	34 (4.78)	12.32 ± 3.531		
Marriage	Unmarried	315 (44.30)	10.32 ± 2.866	0.802	0.449
	Married	383 (53.87)	10.51 ± 3.310		
	Divorce	13 (1.83)	11.31 ± 3.924		
Educational level	Junior high school and below	210 (29.54)	10.03 ± 2.750	-2.412	0.016
	Bachelor degree or above	501 (70.46)	10.61 ± 3.267		
Working life	≤5 years	340 (47.82)	10.20 ± 3.034	4.775	0.003
	6~9 years	132 (18.57)	10.61 ± 3.041		
	10~19 years	215 (30.24)	10.47 ± 3.194		
	≥20 years	24 (3.34)	12.63 ± 3.681		
Professional qualifications	Primary	571 (80.31)	10.37 ± 3.062	-1.125	0.261
	Middle grade or above	140 (19.69)	10.71 ± 3.402		
Average monthly income	<5000 yuan	190 (26.72)	10.93 ± 2.748	4.314	0.014
	5000~10000 yuan	427 (60.06)	10.36 ± 3.151		
	>10000 yuan	94 (13.22)	9.82 ± 3.631		
Night shift factor					
Average duration of each night shift	≤8 hours	480 (67.51)	10.26 ± 3.246	6.269	0.002
	9~16 hours	206 (28.97)	10.61 ± 2.747		
	≥17 hours	25 (3.52)	12.44 ± 3.229		
Number of consecutive days per night shift	1 days	80 (11.25)	9.81 ± 3.094	2.265	0.105
	2 days	407 (57.24)	10.43 ± 3.100		
	≥3 days	224 (31.50)	10.68 ± 3.185		
Average weekly night shift frequency	Less than or equal to once	271 (38.12)	9.80 ± 3.180	10.080	0.000
	2 times	346 (48.66)	10.75 ± 2.945		
	Greater than or equal to 3 times	94 (13.22)	11.15 ± 3.360		
Average night shift interval days	≤5 days	413 (58.09)	10.98 ± 3.088	15.001	0.000
	6~9 days	256 (36.01)	9.71 ± 3.067		
	≥10 days	42 (5.91)	9.60 ± 2.947		
Quality of make-up sleep the day before night shift	Good	73 (10.27)	7.73 ± 1.960	63.966	0.000
	Generally	509 (71.59)	10.32 ± 2.968		
	Poor	129 (18.14)	12.45 ± 2.995		
Quality of make-up sleep the day after night shift	Good	90 (12.66)	7.81 ± 2.087	67.086	0.000
	Generally	511 (71.87)	10.45 ± 2.984		
	Poor	110 (15.47)	12.55 ± 2.914		
Night shift in life	≤5 years	367 (51.62)	10.24 ± 2.967	1.919	0.148
	6~9 years	131 (18.42)	10.46 ± 3.097		
	≥10 years	213 (29.96)	10.77 ± 3.406		
Life rules					
Is the daily routine regular	Very regular	43 (6.05)	7.93 ± 3.390	36.343	0.000
	More regular	305 (42.90)	9.76 ± 3.060		
	Less irregular	241 (33.90)	10.73 ± 2.615		
	Very irregular	122 (17.16)	12.46 ± 2.966		
Exercise every day	No	318 (44.73)	10.56 ± 3.062	0.939	0.348
	Yes	393 (55.27)	10.34 ± 3.188		
Daily diet	Very regular	33 (4.64)	9.85 ± 3.914	21.460	0.000
	More regular	385 (54.15)	9.69 ± 3.049		
	Less irregular	236 (33.19)	11.33 ± 2.826		
	Very irregular	57 (8.02)	12.14 ± 2.825		

PSQI, Pittsburgh sleep quality index

### PSQI according to the participants’ characteristics

Nurses had a mean (SD) PSQI score of 10.44 (± 3.13). PSQI scores differed significantly based on the participants’ characteristics except for marriage, professional qualifications, number of consecutive days per night shift, night shift in life and daily exercise. Nurses who were female (*p* < 0.05), were over 40 years of age (*p* < 0.01) and had bachelor degree or above (*p* < 0.05) and had an average monthly income of <5,000 yuan (*p* < 0.05) were more likely to show lower sleep quality than were the other groups. With regard to work-related characteristics, nurses who had a working period of ≥20 years (*p* < 0.01), an average duration of over 17 h for each night shift (*p* < 0.01), an average weekly night shift frequency of ≥3 times (*p* < 0.01), an average night shift interval of <5 days (*p* < 0.01) and poor quality of make-up sleep during night shifts (*p* < 0.01) reported worse sleep quality than did the other groups. With regard to life-related characteristics, those with very irregular daily routine (*p* < 0.01) and daily diet (*p* < 0.01) showed poorer sleep quality than did the other groups (**Table 1**).

### Factors predicting sleep quality

Generalized linear regression analysis was conducted to determine factors predictive of sleep quality. The potential influencing factors that showed statistical significance during the t-test and ANOVA were included in the generalized linear analysis. Our results showed that education level, years of working experience, quality of make-up sleep before and after night shifts, daily routine and diet were the primary factors affecting sleep quality (*p* < 0.01) (**Table 2**).

**Table 2:** Multivariate analysis of factors affecting sleep quality among night-shift nurses (n=711).

Variables	Group	β	SE	Waldχ^2^	P value	95%CI	
						Lower limit	Upper limit
General demographic factors							
Gender	Male	-0.268	0.3429	0.611	0.434	-0.940	0.404
	Female (reference)						
Age	18~29	-0.808	0.7630	1.121	0.290	-2.303	0.688
	30~39	-0.549	0.6688	0.675	0.411	-1.860	0.761
	≥40 (reference)						
Educational level	Junior high school and below	-0.671	0.2317	8.398	0.004	-1.125	-0.217
	Bachelor degree or above (reference)						
Working life	≤5 years	-2.170	0.881	5.970	0.015	-3.911	-0.429
	6~9 years	-2.350	0.8674	7.339	0.007	-4.050	-0.650
	10~19 years	-2.195	0.7843	7.831	0.005	-3.732	-0.658
	≥20 years (reference)						
Average monthly income	<5000 yuan	0.491	0.3669	1.788	0.181	-0.229	1.210
	5000~10000 yuan	0.290	0.3192	0.826	0.363	-0.336	0.851
	>10000 yuan (reference)						
Night shift factor							
Average duration of each night shift	≤8 hours	-0.959	0.5425	3.127	0.077	-2.022	0.104
	9~16 hours	-1.028	0.5557	3.421	0.064	-2.117	0.061
	≥17 hours (reference)						
Average weekly night shift frequency	Less than or equal to once	-0.043	0.3452	0.015	0.902	-0.719	0.634
	2 times	0.236	0.3136	0.566	0.452	-0.379	0.851
	Greater than or equal to 3 times (reference)						
Average night shift interval days	≤5 days	0.820	0.4540	3.264	0.071	-0.070	1.710
	6~9 days	0.166	0.4421	0.140	0.708	-0.701	1.032
	≥10 days (reference)						
Quality of make-up sleep the day before night shift	Good	-2.233	0.4724	22.342	0.000	-3.159	-1.307
	Generally	-0.992	0.3070	10.433	0.001	-1.594	-0.390
	Poor (reference)						
Quality of make-up sleep the day after night shift	Good	-2.283	0.4576	24.887	0.000	-3.180	-1.386
	Generally	-1.056	0.3261	10.495	0.001	-1.695	-0.417
	Poor (reference)						
Life rules							
Is the daily routine regular	Very regular	-3.194	0.4969	41.309	0.000	-4.167	-2.220
	More regular	-1.398	0.3125	20.031	0.000	-2.011	-0.786
	Less irregular	-1.116	0.2982	14.010	0.000	-1.701	-0.532
	Very irregular (reference)						
Daily diet	Very regular	-0.511	0.6123	0.696	0.404	-1.711	0.689
	More regular	-1.115	0.4021	7.695	0.006	-1.903	-0.327
	Less irregular	-0.343	0.3909	0.769	0.380	-1.109	0.423
	Very irregular (reference)						

SE, Standard error; 95% CI, 95% confidence interval.

### Correlation between sleep quality and chronic fatigue and occupational burnout

Most nurses (n = 642, 90.1%) reported poor sleep quality, which is supported by our study’s finding of a global sleep quality score of ≥7. The mean (SD) of each component of sleep quality was as follows: a sleep quality, 1.56 (± 0.755); sleep latency, 1.08 (± 0.734); sleep duration, 1.44 (± 0.808); sleep efficiency, 2.89 (± 0.502); sleep disturbance, 1.25 (± 0.683); medications to sleep, 0.33 (± 0.778); and daytime dysfunctions, 1.89 (± 0.879) (**Table 3**). Nurses had a mean (SD) BFI score of 5.05 (± 2.30). The mean (SD) of each component of the BFI was as follows: comprehensive fatigue degree, 5.66 (± 2.28) and Comprehensive fatigue effect degree, 4.74 (± 2.45). Specifically, nurse fatigue was significantly positively correlated with subjective sleep quality, sleep latency, sleep duration, sleep disturbance, medications to sleep, daytime dysfunctions and global sleep quality (*p* < 0.01). However, sleep efficiency was significantly negatively correlated (*p* < 0.01) with fatigue.

**Table 3:** Descriptive statistics and correlation analysis of variables.

Variables	M	SD	Variables		
			BFI	Comprehensive fatigue level	Comprehensive fatigue effect
PSQI	10.44	3.132	0.522^**^	0.532^**^	0.486^**^
Sleep quality	1.56	0.755	0.420^**^	0.433^**^	0.388^**^
Sleep latency	1.08	0.734	0.364^**^	0.369^**^	0.341^**^
Sleep duration	1.44	0.808	0.272^**^	0.287^**^	0.249^**^
Sleep efficiency	2.89	0.502	-0.149^**^	-0.142^**^	-0.143^**^
Sleep disturbance	1.25	0.683	0.491^**^	0.467^**^	0.473^**^
Medications to sleep	0.33	0.778	0.184^**^	0.190^**^	0.171^**^
Daytime dysfunctions	1.89	0.879	0.485^**^	0.501^**^	0.448^**^

PSQI, Pittsburgh sleep quality index; BFI, Brief Fatigue Inventory-Chinese Version; M, mean; SD, standard deviation; *P<0.05, **P<0.01.

## Discussion

### Generally poor sleep quality among night-shift nurses

The results presented herein showed that the prevalence rate of poor sleep quality among night-shift nurses reached as high as 90.1%, which was much higher than that reported in similar previous studies. A large survey from Norway showed that the prevalence of symptoms of shift work disorder (e.g. difficulties with sleeping, excessive sleepiness and insomnia) among night-shift nurses was around 40% (24). Another study suggested that the prevalence of poor sleep quality was 62.11% in nurses working consecutive night shifts and 55.75% in nurses having worked night shifts before (3), which may have been related to nurses coming from hospitals at different regions. Various hospitals have inconsistent time requirements for various shifts, with the main models of shift work consisting of two shifts (day/night shift) and three shifts (day/morning–evening/night shift) (7); hence, the incidence of poor sleep quality can vary among different night shift modes. The internal mechanism by which shift work affects sleep is believed to be environmental stress caused by shift work, which affects the suprachiasmatic nuclei of the hypothalamus and interferes with the mechanism that synchronises the endogenous biological clock with the external environment (25), thereby desynchronizing the body’s sleep-wake routines. Night shifts exacerbate the severity of circadian disruption, with frequent circadian rhythm disruptions hindering complete rest of the body and the reestablishment of normal circadian rhythms. Thus, the prevalence rate of poor sleep quality among night-shift nurses has remained relatively high.

### Analysis of factors influencing poor sleep quality among night-shift nurses

Our results showed that education level, years of working experience, quality of make-up sleep before and after night shifts, daily routine and diet were the primary factors affecting sleep quality (*p* < 0.01).

First, we found that nurses with higher education levels (undergraduate or higher) were at higher risk for developing sleep problems after working night shifts. With the improvement of education level and perfection of the nursing education system, nurses with higher education levels receive more complete nursing knowledge, which increase their nursing knowledge reserve and skill level. They pay more attention to inner feelings, pursue spiritual satisfaction and have higher requirements for career development. Compared to nurses with bachelor’s degree below, whose main task is clinical work, nurses with higher education levels undertake more management, research, and teaching work, and may not receive sufficient rest after night shifts. And the incidence of poor sleep quality caused by stress and anxiety increased.

Second, night-shift nurses who worked longer hours were at higher risk of developing sleep problems, consistent with the research findings of Dong et al. (26). The Chinese Ministry of Health classifies nurses into registered, primary, intermediate, deputy senior and senior nurses according to years of service, working ability and capacity for scientific research (26). Nurses with longer service years often bear higher work tasks and responsibilities; therefore, pressure during night shifts would be greater. They often expose themselves to more sleep problems, which can be complicated by the increase in physical age and inability to adapt to the intensity of night shift work.

Third, the quality of make-up sleep before and after night shifts has been identified as the main factor affecting sleep. Previously, few studies had explored the impact of pre-night-shift sleep quality on night-shift nurses. One study reported that nurses had an average of 414 min or just below 7 h, of sleep before a work day and 497 min or just over 8 h, before a nonwork day, which may impact their health and performance (27). During the day, melatonin is suppressed by daylight and promotes wakefulness; therefore, nurses who sleep during the day are faced with sleep difficulties (28). Furthermore, the duration of rest after the end of night shifts is crucial for the sleep quality of night-shift nurses. In fact, nurses who have sufficient time to rest can better recover from fatigue and compensate for insufficient sleep. In contrast, nurses who have insufficient rest periods after night shifts may not obtain adequate rest, resulting in ongoing sleep deprivation and accumulation of fatigue (16). Nurses may receive training, assessments or meetings after night shifts and may not be able to replenish their sleep in a timely manner. Sleep reduction can also be attributed to family responsibilities, such as accompanying their spouses and taking care of children or the elderly, which may prevent night-shift nurses from supplementing their sleep time (27). Selective deprivation of fast-wave sleep among night-shift nurses resulted in rebound during fast-wave sleep, leading to a significant decline in sleep quality.

Fourthly, the daily routine of night-shift nurses can affect their sleep quality. Previous studies have shown that female nurses working rotating shifts are required to change their daily routines often, which affects their biological circadian rhythms and could cause sleep problems and fatigue (29). Studies have shown that those working night shifts are also exposed to disrupted circadian rhythm, stress, lack of physical activity and sleep debt, which can impact their health (30, 31). However, a study in the United States found no differences in sleep and diet quality between nurses on day and night shifts. However, our findings revealed that daily routines are crucial for the sleep quality of night-shift nurses, which contradict the results of the aforementioned study.

Finally, we found that the daily diet also was a primary factors affecting sleep quality. Working as a nurse creates an unpredictable environment that can affect meal patterns (32). Prior studies have indicated that a majority of nurses had poor diet, health and physical activity patterns (33, 34). Nurses tend to skip lunch and breaks to complete their work, which promotes irregular meal patterns (35). Several nurses are less informed about the important relationship between nutrition and exercise, which are both vital for successfully maintaining a healthy weight (36). Unusual working hours leads to behavioral changes, such as altered diet and weight gain, resulting in inadequate sleep. Consequently, poor sleep may negatively impact physical health by promoting unhealthy eating habits, obesity, metabolic syndrome and diabetes, creating a vicious cycle (37).

### Relationship between sleep quality and chronic fatigue among night-shift nurses

Another common complaint of night-shift nurses is fatigue, a symptom characterized by feelings of tiredness and lack of energy that is closely associated with sleepiness (38). Persistent poor sleep quality may increase fatigue, which in turn can negatively impact patient and staff safety (39). Growing evidence has shown that long working hours and shift work are associated with errors in the delivery of patient care and vehicle crashes on the commute (40). Our results also found a strong correlation between sleep quality and chronic fatigue among night-shift nurses. This may be related to migraines, people suffering from migraines are significantly more likely to suffer from poor sleep quality, insomnia and night-time fatigue (41).

After finishing their night shift, nurses still take on many household chores, such as cleaning, cooking, laundry and caring for family members (42), which prevent them from making up for their sleep within a short period of time. The deprivation of normal sleep time seriously affects sleep quality among nurses. Long-term sleep deprivation can cause emotional disorders, behavioral disorders and various diseases. Insufficient nurse staffing shortens the rotation period of night shifts for each nurse. Frequent night shifts within a short period of time increases not only the workload of nurses but also their stress levels (43). The fast-paced work environment of nurses can easily place them in a state of stress, which can alter their immune, endocrine, neurobiochemical functions, among others. Frequent night shifts also increase physical fatigue, causing occupational burnout, decreased work enthusiasm and alertness (44) and increased occurrence of nursing errors, which negatively impact the quality of nursing care.

### Suggestions for clinical practice

Shift nurses can take some measures to improve their sleep quality and health, such as regular and reasonable exercise, taking naps during the night shift, avoiding consumption of high-fat foods after midnight, wearing dark sunglasses when driving home, sleeping in a room with blackout curtains and minimizing morning sunlight (7). Evidence suggests that inhalation aromatherapy significantly improves shift-work nurses’ sleep quality (45). In addition, appropriate management of nurses should involve a people-oriented concept, improving the work system, reasonable arranging work, reducing occupational burnout, preventing overwork, strengthening daily nursing care, promoting regular physical examination, paying attention to nurses’ emotional state, securing the physical and mental health of nurses, improving work enthusiasm and work efficiency, reducing errors and improving the quality of nursing. Studies focusing on sleep education and training for shift working nurses have been scarce (46); nonetheless, nursing managers need to promote health education and awareness of sleep health among shift nurses (47).

### Strengths and limitations

This study has several limitations. First, our sample size was quite small and was limited to the nurse population in the Sichuan Province, which limits the generalizability of our findings. Second, given the cross-sectional nature of this study, certain limitations in causal inference are unavoidable. Third, this study used convenience sampling for participant recruitment and did not perform stratified sampling for different hospital levels. Fourthly, we use a scale to assess sleep problems, which has a certain degree of subjectivity. In the future, objective assessment of sleep disorders can be combined with polysomnography (PSG). And multi-center longitudinal studies on sleep-promoting interventions for night-shift nurses are warranted to improve nurses’ occupational health.

## Conclusions

The current study found that the prevalence of poor sleep quality among night-shift nurses reached as high as 90.1%. Moreover, we found that education level, years of working life, quality of make-up sleep before and after night shifts, daily routine and daily diet were exposure risk factors that affect nurses’ sleep quality. Poor sleep among night-shift nurses has been strongly correlated with chronic fatigue. Interventions that promote sleep quality among night-shift nurses at the individual and managerial levels are needed to improve their overall health.

## Data Availability

The raw data supporting the conclusions of this article will be made available by the authors, without undue reservation.

## References

[1] Dai C Qiu H Huang Q Hu P Hong X Tu J. The effect of night shift on sleep quality and depressive symptoms among Chinese nurses. Neuropsychiatr Dis Treat. (2019) 15:435–40. doi: 10.2147/NDT.S190689 PMC6369837 30799922

[2] Bin-Bin SU Juan DU Jin-Zhong J Yuan-Yuan W Zheng-Wei J Chi Z. Study on the current situation and allocation equity of China's nursing human resources. Chin J Health Policy. (2018) 11:56–61. doi: 10.3969/j.issn.1674-2982.2018.12.010

[3] Huang Q Tian C Zeng XT. Poor sleep quality in nurses working or having worked night shifts: A cross-sectional study. Front Neurosci. (2021) 15:638973. doi: 10.3389/fnins.2021.638973 34413721 PMC8369413

[4] Cheng YH Lech M Wilkinson RH. Simultaneous sleep stage and sleep disorder detection from multimodal sensors using deep learning. Sensors (Basel). (2023) 23(7):3468. doi: 10.3390/s23073468 PMC10099216 37050528

[5] Brum MCB Senger MB Schnorr CC Ehlert LR Rodrigues TDC. Effect of night-shift work on cortisol circadian rhythm and melatonin levels. Sleep Sci. (2022) 15:143–8. doi: 10.5935/1984-0063.20220034 PMC9210564 35755906

[6] Zhang H Wang J Zhang S Tong S Hu J Che Y. Relationship between night shift and sleep problems, risk of metabolic abnormalities of nurses: a 2 years follow-up retrospective analysis in the National Nurse Health Study (NNHS). Int Arch Occup Environ Health. (2023) 96:1361–71. doi: 10.1007/s00420-023-02014-2 PMC10635907 37874403

[7] Feng HL Qi XX Xia CL Xiao SQ Fan L. Association between night shift and sleep quality and health among Chinese nurses: A cross-sectional study. J Nurs Manage. (2021) 29:2123–31. doi: 10.1111/jonm.13351 33908108

[8] Alreshidi SM Rayani AM. The correlation between night shift work schedules, sleep quality, and depression symptoms. Neuropsychiatr Dis Treat. (2023) 19:1565–71. doi: 10.2147/NDT.S421092 PMC10335288 37440839

[9] Park E Lee HY Park CS. Association between sleep quality and nurse productivity among Korean clinical nurses. J Nurs Manage. (2018) 26:1051–8. doi: 10.1111/jonm.2018.26.issue-8 29855101

[10] Chang WP Peng YX. Influence of rotating shifts and fixed night shifts on sleep quality of nurses of different ages: a systematic literature review and meta-analysis. Chronobiol Int. (2021) 38:1384–96. doi: 10.1080/07420528.2021.1931273 34056959

[11] Beebe D Chang JJ Kress K Mattfeldt-Beman M. Diet quality and sleep quality among day and night shift nurses. J Nurs Manage. (2017) 25:549–57. doi: 10.1111/jonm.2017.25.issue-7 28695685

[12] Imes CC Tucker SJ Trinkoff AM Chasens ER Weinstein SM Dunbar-Jacob J. Wake-up call: night shifts adversely affect nurse health and retention, patient and public safety, and costs. Nurs Adm Q. (2023) 47:E38–e53. doi: 10.1097/NAQ.0000000000000595 37643236

[13] Alfonsi V Scarpelli S Gorgoni M Pazzaglia M Giannini AM De Gennaro L. Sleep-related problems in night shift nurses: towards an individualized interventional practice. Front Hum Neurosci. (2021) 15:644570. doi: 10.3389/fnhum.2021.644570 33796014 PMC8007770

[14] James L James SM Wilson M Brown N Dotson EJ Dan Edwards C. Sleep health and predicted cognitive effectiveness of nurses working 12-hour shifts: an observational study. Int J Nurs Stud. (2020) 112:103667. doi: 10.1016/j.ijnurstu.2020.103667 32593476

[15] Leyva-Vela B Jesús Llorente-Cantarero F Henarejos-Alarcón S Martínez-Rodríguez A. Psychosocial and physiological risks of shift work in nurses: a cross-sectional study. Cent Eur J Public Health. (2018) 26:183–9. doi: 10.21101/cejph.a4817 30419619

[16] Yuan MZ Fang Q. Latent class analysis of the sleep quality of night shift nurses and impact of shift-related factors on the occupational stress and anxiety. J Adv Nurs. (2024) 80:2772–84. doi: 10.1111/jan.16067 38235926

[17] Mohamed AZ Andersen T Radovic S Del Fante P Kwiatek R Calhoun V. Objective sleep measures in chronic fatigue syndrome patients: A systematic review and meta-analysis. Sleep Med Rev. (2023) 69:101771. doi: 10.1016/j.smrv.2023.101771 36948138 PMC10281648

[18] Li J Chen X Han X Zhang G. Spatiotemporal matching between medical resources and population ageing in China from 2008 to 2017. BMC Public Health. (2020) 20:845. doi: 10.1186/s12889-020-08976-z 32493251 PMC7268461

[19] Liu XC Tang MQ Hu L Wang AZ Wu HX Zhao GF. Reliability and validity of the Pittsburgh sleep quality index. Chin J Psychiatry. (1996) 29:103–7.

[20] Boned-Galán Á López-Ibort N Gascón-Catalán A. Sleep disturbances in nurse managers during the early and late stages of the COVID-19 pandemic. Front Psychol. (2022) 13:1070355. doi: 10.3389/fpsyg.2022.1070355 36591079 PMC9801981

[21] Chen R Fang P Tan L Li J Yu L. Factors associated with sleep quality among nurse managers during regular prevention and control of the COVID-19 in China: a cross-sectional study. BMC Nurs. (2022) 21:363. doi: 10.1186/s12912-022-01149-w 36536398 PMC9761616

[22] Mollayeva T Thurairajah P Burton K Mollayeva S Shapiro CM Colantonio A. The Pittsburgh sleep quality index as a screening tool for sleep dysfunction in clinical and non-clinical samples: A systematic review and meta-analysis. Sleep Med Rev. (2016) 25:52–73. doi: 10.1016/j.smrv.2015.01.009 26163057

[23] Wang XS Hao XS Wang Y Guo H Jiang YQ Mendoza TR. Validation study of the Chinese version of the Brief Fatigue Inventory (BFI-C). J Pain Symptom Manage. (2004) 27:322–32. doi: 10.1016/j.jpainsymman.2003.09.008 15050660

[24] Flo E Pallesen S Magerøy N Moen BE Grønli J Hilde Nordhus I. Shift work disorder in nurses–assessment, prevalence and related health problems. PloS One. (2012) 7:e33981. doi: 10.1371/journal.pone.0033981 22485153 PMC3317447

[25] Luca G Van Den Broecke S. Circadian rhythm sleep disorders: clinical picture, diagnosis and treatment. Rev Med Suisse. (2020) 16:1237–42. 32558452

[26] Dong H Zhang Q Sun Z Sang F Xu Y. Sleep problems among Chinese clinical nurses working in general hospitals. Occup Med (Lond). (2017) 67:534–9. doi: 10.1093/occmed/kqx124 29016953

[27] Stimpfel AW Fatehi F Kovner C. Nurses' sleep, work hours, and patient care quality, and safety. Sleep Health. (2020) 6:314–20. doi: 10.1016/j.sleh.2019.11.001 31838021

[28] Chaiard J Deeluea J Suksatit B Songkham W Inta N Stone TE. Sleep disturbances and related factors among nurses. Nurs Health Sci. (2019) 21:470–8. doi: 10.1111/nhs.12626 31317652

[29] Chang WP Li HB. Influence of shift work on rest-activity rhythms, sleep quality, and fatigue of female nurses. Chronobiol Int. (2022) 39:557–68. doi: 10.1080/07420528.2021.2005082 34906006

[30] Kunst JR Løset GK Hosøy D Bjorvatn B Moen BE Magerøy N. The relationship between shift work schedules and spillover in a sample of nurses. Int J Occup Saf Ergon. (2014) 20:139–47. doi: 10.1080/10803548.2014.11077030 24629875

[31] Lowden A Moreno C Holmbäck U Lennernäs M Tucker P. Eating and shift work - effects on habits, metabolism and performance. Scand J Work Environ Health. (2010) 36:150–62. doi: 10.5271/sjweh.2898 20143038

[32] Nahm ES Warren J Friedmann E Brown J Rouse D Kyung Park B. Implementation of a participant-centered weight management program for older nurses: A feasibility study. Online J Issues Nurs. (2014) 19:4. doi: 10.3912/OJIN.Vol19No03Man04 26824152

[33] Sveinsdóttir H Gunnarsdóttir HK. Predictors of self-assessed physical and mental health of Icelandic nurses: results from a national survey. Int J Nurs Stud. (2008) 45:1479–89. doi: 10.1016/j.ijnurstu.2008.01.007 18329648

[34] Zapka JM Lemon SC Magner RP Hale J. Lifestyle behaviours and weight among hospital-based nurses. J Nurs Manage. (2009) 17:853–60. doi: 10.1111/j.1365-2834.2008.00923.x PMC2760042 19793242

[35] King KA Vidourek R Schwiebert M. Disordered eating and job stress among nurses. J Nurs Manage. (2009) 17:861–9. doi: 10.1111/j.1365-2834.2009.00969.x 19793243

[36] Gabel Speroni K. Designing exercise and nutrition programs to promote normal weight maintenance for nurses. Online J Issues Nurs. (2014) 19:6. doi: 10.3912/OJIN.Vol19No03Man06 26824154

[37] Jung G Oh J. The relationship between childhood trauma, eating disorders, and sleep quality among registered hospital nurses in South Korea. Healthcare (Basel). (2020) 8(4):490. doi: 10.3390/healthcare8040490 PMC7711980 33212992

[38] Smith-Miller CA Shaw-Kokot J Curro B Jones CB. An integrative review: fatigue among nurses in acute care settings. J Nurs Adm. (2014) 44:487–94. doi: 10.1097/NNA.0000000000000104 25148403

[39] Sagherian K Clinton ME Abu-Saad Huijer H Geiger-Brown J. Fatigue, work schedules, and perceived performance in bedside care nurses. Workplace Health Saf. (2017) 65:304–12. doi: 10.1177/2165079916665398 27872407

[40] Caruso CC. Negative impacts of shiftwork and long work hours. Rehabil Nurs. (2014) 39:16–25. doi: 10.1002/rnj.107 23780784 PMC4629843

[41] Waliszewska-Prosół M Nowakowska-Kotas M Chojdak-Łukasiewicz J Budrewicz S. Migraine and sleep-an unexplained association? Int J Mol Sci. (2021) 22(11):5539. doi: 10.3390/ijms22115539 PMC8197397 34073933

[42] Chang WP Wang CH. Influence of sleep fragmentation and fatigue on turnover of female nurses working rotating shifts. J Clin Nurs. (2022) 31:3573–83. doi: 10.1111/jocn.v31.23-24 34957611

[43] Knupp AM Patterson ES Ford JL Zurmehly J Patrick T. Associations among nurse fatigue, individual nurse factors, and aspects of the nursing practice environment. J Nurs Adm. (2018) 48:642–8. doi: 10.1097/NNA.0000000000000693 30431518

[44] Min A Hong HC Son S Lee T. Sleep, fatigue and alertness during working hours among rotating-shift nurses in Korea: An observational study. J Nurs Manage. (2021) 29:2647–57. doi: 10.1111/jonm.13446 34351017

[45] Kang J Noh W Lee Y. Sleep quality among shift-work nurses: A systematic review and meta-analysis. Appl Nurs Res. (2020) 52:151227. doi: 10.1016/j.apnr.2019.151227 31902652

[46] Hittle BM Hils J Fendinger SL Wong IS. A scoping review of sleep education and training for nurses. Int J Nurs Stud. (2023) 142:104468. doi: 10.1016/j.ijnurstu.2023.104468 37080122 PMC10180237

[47] Sun Q Ji X Zhou W Liu J. Sleep problems in shift nurses: A brief review and recommendations at both individual and institutional levels. J Nurs Manage. (2019) 27:10–8. doi: 10.1111/jonm.2019.27.issue-1 30171641

